# Comprehensive Registry of Esophageal Cancer in Japan, 2009

**DOI:** 10.1007/s10388-016-0531-y

**Published:** 2016-03-29

**Authors:** Yuji Tachimori, Soji Ozawa, Hodaka Numasaki, Mitsuhiro Fujishiro, Hisahiro Matsubara, Tsuneo Oyama, Masayuki Shinoda, Yasushi Toh, Harushi Udagawa, Takashi Uno, The Registration Committee for Esophageal Cancer of the Japan Esophageal Society

**Affiliations:** Esophageal Surgery Division, National Cancer Center Hospital, 5-1-1 Tsukiji, Chuo-ku, Tokyo, 104-0045 Japan; Department of Gastroenterological Surgery, Tokai University School of Medicine, Isehara, Japan; Department of Medical Physics and Engineering, Osaka University Graduate School of Medicine, Osaka, Japan; Department of Endoscopy and Endoscopic Surgery, Graduate School of Medicine, University of Tokyo, Tokyo, Japan; Department of Frontier Surgery, Graduate School of Medicine, Chiba University, Chiba, Japan; Department of Gastroenterology, Saku General Hospital, Nagano, Japan; Department of Thoracic Surgery, Aichi Cancer Center Hospital, Aichi, Japan; Department of Gastroenterological Surgery, National Kyushu Cancer Center, Fukuoka, Japan; Department of Gastroenterological Surgery, Toranomon Hospital, Tokyo, Japan; Department of Radiology, Graduate School of Medicine, Chiba University, Chiba, Japan

## Preface 2009

We deeply appreciate the great contributions of many physicians in the registry of esophageal cancer cases. The Comprehensive Registry of Esophageal Cancer in Japan, 2009 was published here, despite some delay. The registry complies with the Act for the Protection of Personal Information. The encryption with a HASH function is used for ‘‘anonymity in an unlinkable fashion’’.

 We briefly summarized the Comprehensive Registry of Esophageal Cancer in Japan, 2009. Japanese Classification of Esophageal Cancer 10th and UICC TNM Classification 6th were used for cancer staging according to the subjected year. A total of 6260 cases were registered from 276 institutions in Japan. Tumor locations were cervical: 4.4 %, upper thoracic: 11.9 %, middle thoracic: 48.0 %, lower thoracic: 27.7 % and EG junction: 6.6 %. Superficial carcinomas (Tis, T1a, T1b) were 36.7 %. As for the histologic type of biopsy specimens, squamous cell carcinoma and adenocarcinoma accounted for 90.5 and 3.8 %, respectively. Regarding clinical results, the 5-year survival rates of patients treated using endoscopic mucosal resection, concurrent chemoradiotherapy, radiotherapy alone, chemotherapy alone, or esophagectomy were 86.2, 27.9, 20.2, 5.8, and 55.9 %, respectively. Esophagectomy was performed in 3844 cases. Concerning the approach used for esophagectomy, 24.9 % of the cases were treated thoracoscopically. The operative mortality (within 30 days after surgery) was 1.01 % and the hospital mortality was 4.76 %.

 We hope that this Comprehensive Registry of Esophageal Cancer in Japan, 2009 will help to improve all aspects of the diagnosis and treatment of esophageal cancer in Japan.

## Contents

**Fig. 1:**
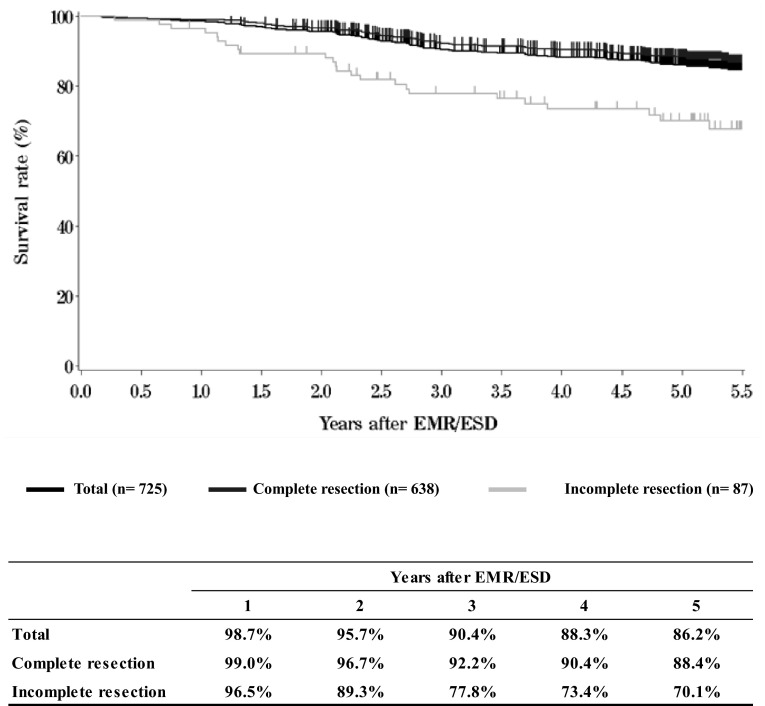
Survival of patients treated with EMR/ESD
  **Figure****2****Survival of patients treated with EMR/ESD according to the pathological depth of tumor invasion (pT)**

I. **Clinical factors of esophageal cancer patients treated in 2009**
  1. Institution-registered cases in 2009
  2. **Patient Background**
    **Table****1****Age and gender**
    **Table****2****Primary treatment**
    **Table****3****Tumor location**
    **Table****4****Histologic types of biopsy specimens**
    **Table****5****Depth of tumor invasion, cT (UICC TNM 6th)**
    **Table****6****Lymph node metastasis, cN (UICC TNM 6th)**
    **Table****7****Distant metastasis, cM (UICC TNM 6th)**
    **Table****8****Clinical stage (UICC TNM 6th)**
II. **Results of endoscopically treated patients in 2009**
  **Table****9****Details of endoscopic treatment**
  **Table****10****Complications of EMR/ESD**
  **Table****11****Pathological depth of tumor invasion of EMR/ESD specimens**
  **Figure****1****Survival of patients treated with EMR/ESD**

**Fig. 2:**
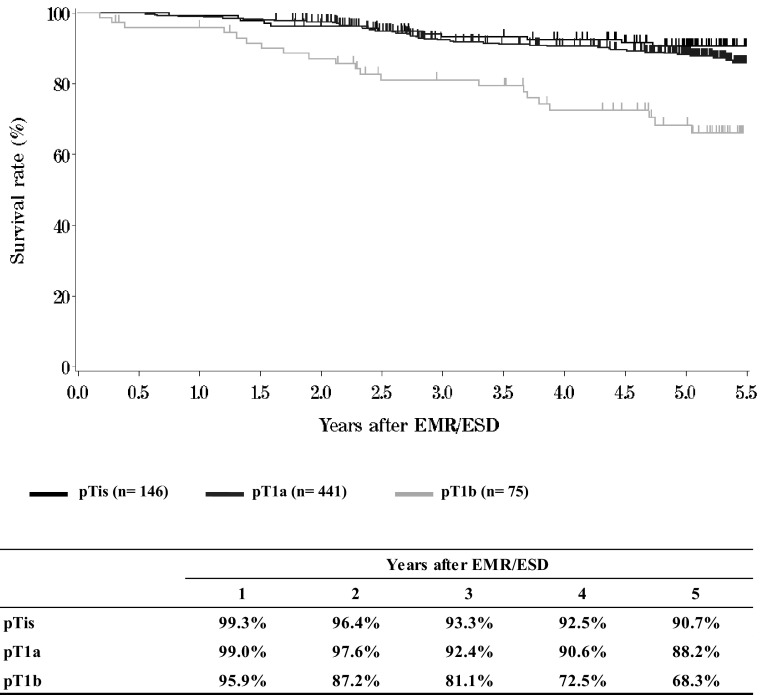
Survival of patients treated with EMR/ESD according to the pathological depth of tumor invasion (pT)
  **Figure****3****Survival of patients treated with EMR/ESD according to the lymphatic and venous invasion**

**Fig. 3:**
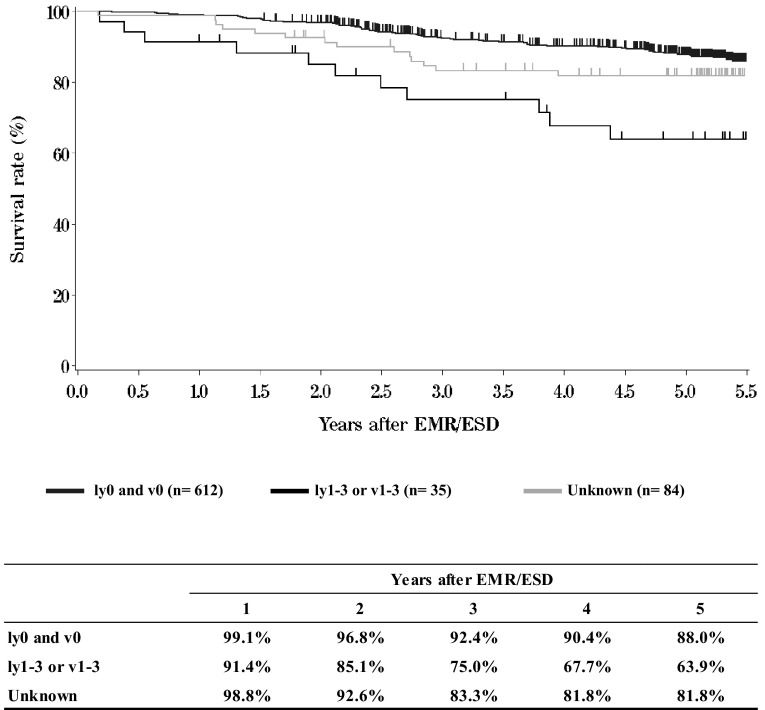
Survival of patients treated with EMR/ESD according to the lymphatic and venous invasion
III. **Results in patients treated with chemotherapy and/or radiotherapy in 2009**
  **Table****12****Dose of radiation (non-surgically treated cases)**
  **Table****13****Dose of radiation (surgically treated cases)**
  **Figure****4****Survival of patients treated with chemotherapy and/or radiotherapy (cStage I-IIA)**

**Fig. 4:**
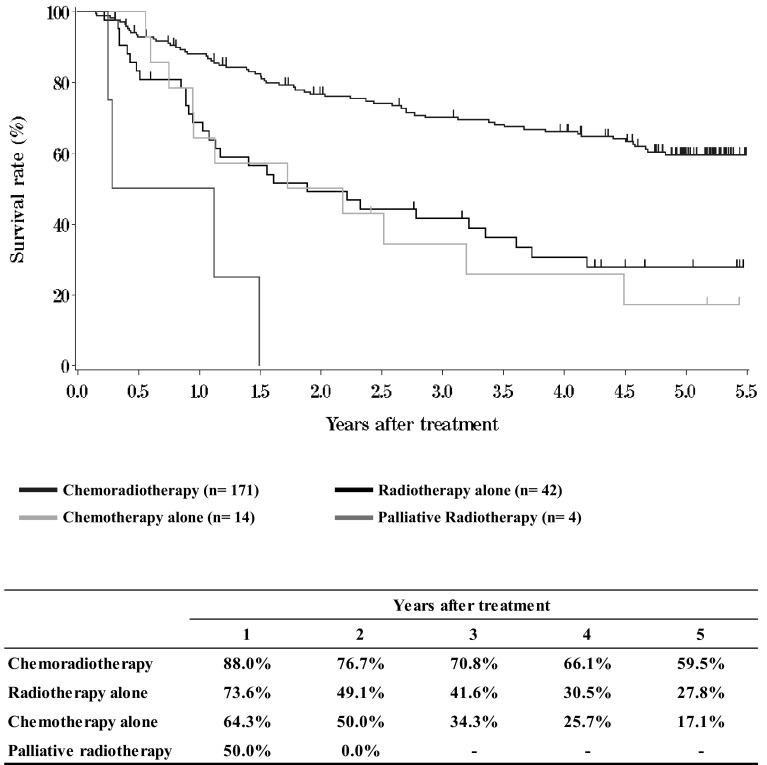
Survival of patients treated with chemotherapy and/or radiotherapy (cStage I-IIA)
  **Figure****5****Survival of patients treated with chemotherapy and/or radiotherapy (cStage IIB-IVB)**

**Fig. 5:**
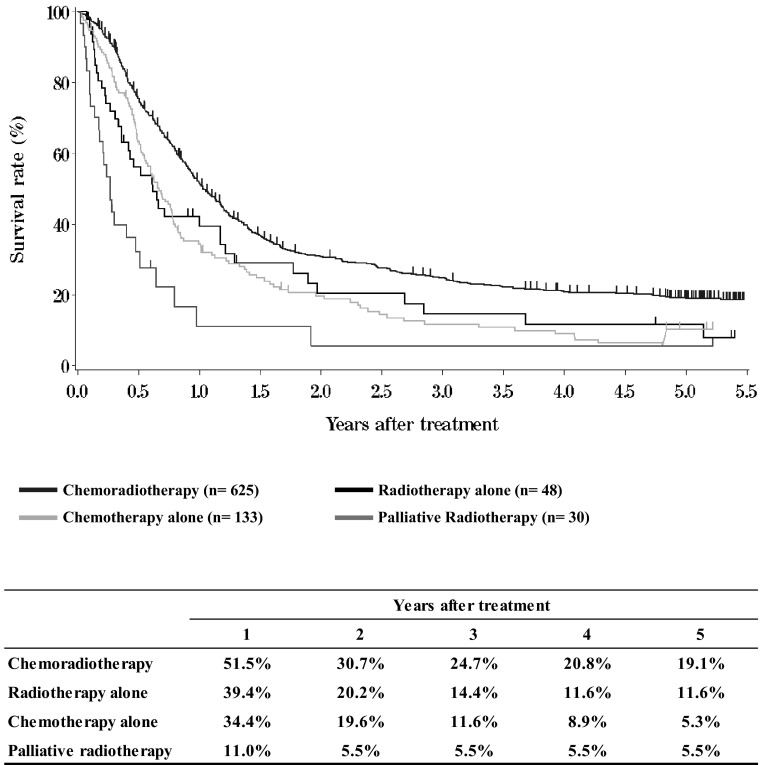
Survival of patients treated with chemotherapy and/or radiotherapy (cStage IIB-IVB)
IV. **Results in patients underwent esophagectomy in 2009**
  **Table****14****Treatment modalities of esophagectomy**
  **Table****15****Tumor location**
  **Table****16****Approaches to tumor resection**
  **Table****17****Video-assisted surgery**
  **Table****18****Fields of lymph node dissection according to the location of the tumor**
  **Table****19****Reconstruction route**
  **Table****20****Organs used for reconstruction**
  **Table****21****Histological classification**
  **Table****22****Depth of tumor invasion, pT (JES 10th)**
  **Table****23****Pathological grading of lymph node metastasis, pN (JES 10th)**
  **Table****24****Numbers of the metastatic nodes**
  **Table****25****Pathological findings of distant organ metastasis, pM (JES 10th)**
  **Table****26****Residual tumor, R**
  **Table****27****Causes of death**
  **Figure****6****Survival of patients who underwent esophagectomy**

**Fig. 6:**
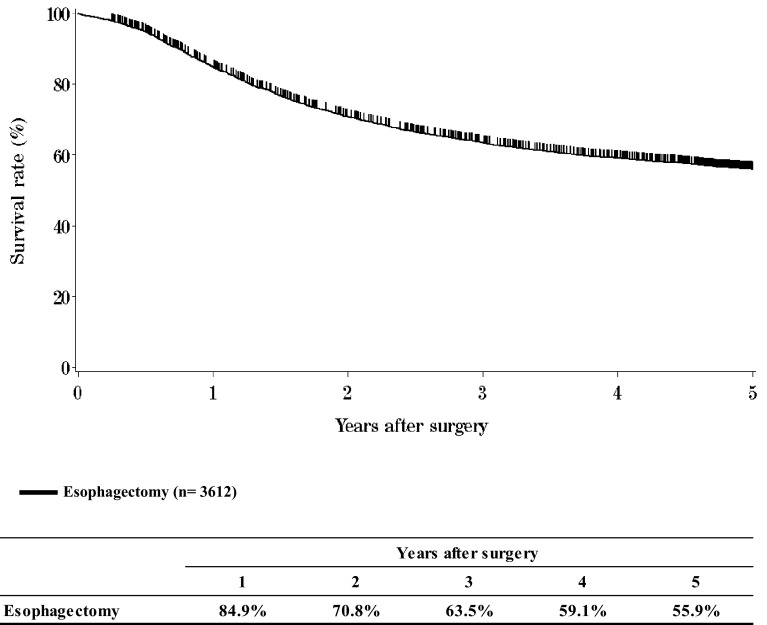
Survival of patients who underwent esophagectomy
  **Figure****7****Survival of patients who underwent esophagectomy according to clinical stage (JES TNM 10th)**

**Fig. 7:**
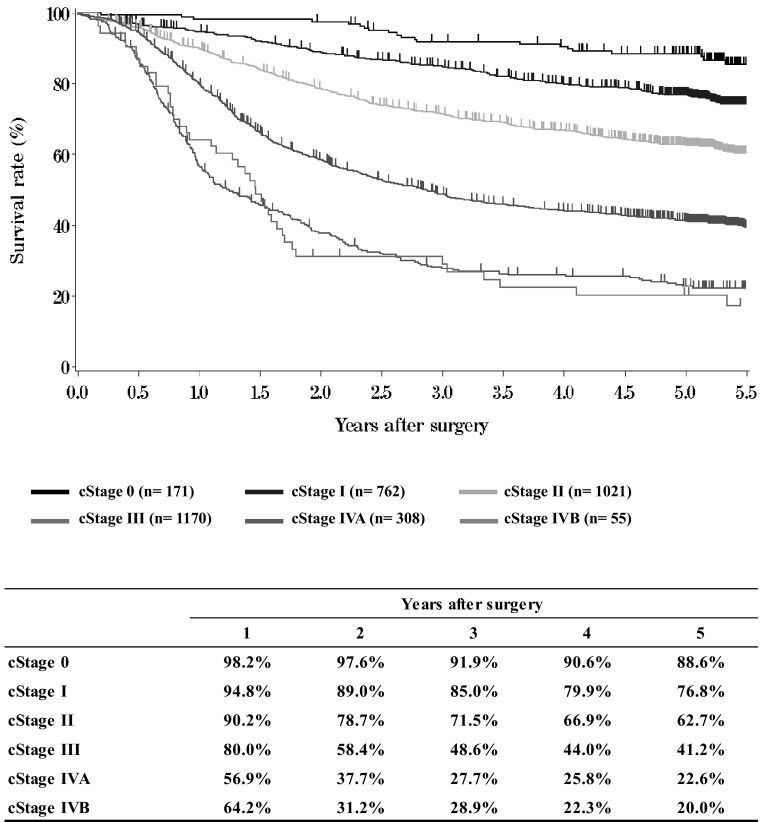
Survival of patients who underwent esophagectomy according to clinical stage (JES TNM 10th)
  **Figure****8****Survival of patients who underwent esophagectomy according to clinical stage (UICC TNM 6th)**

**Fig. 8:**
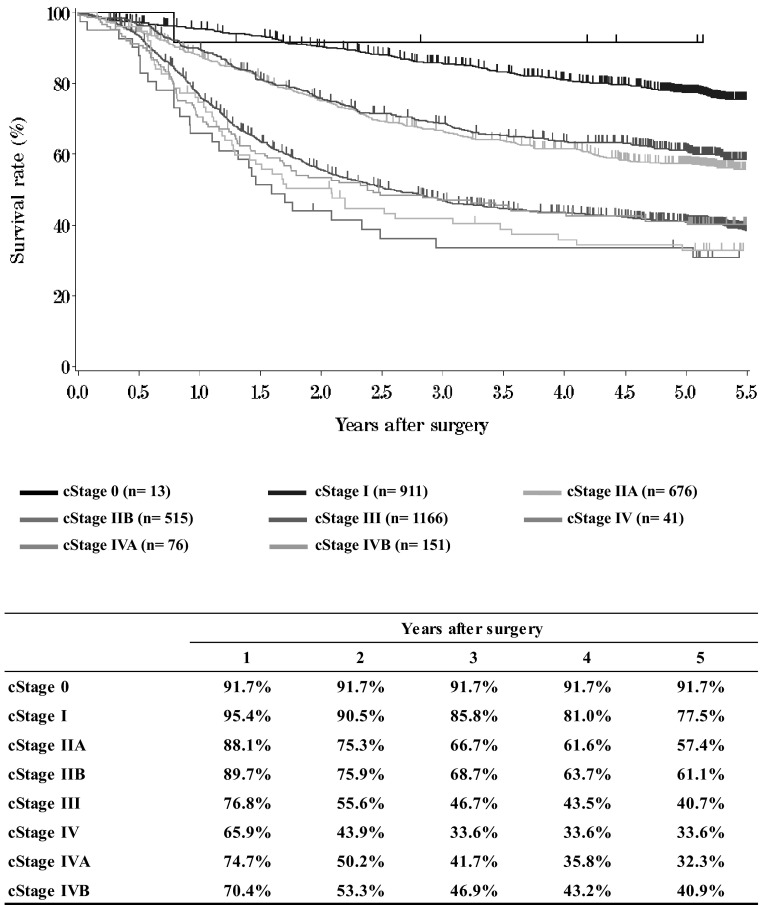
Survival of patients who underwent esophagectomy according to clinical stage (UICC TNM 6th)
  **Figure****9****Survival of patients who underwent esophagectomy according to the depth of tumor invasion (JES 10th: pT)**

**Fig. 9:**
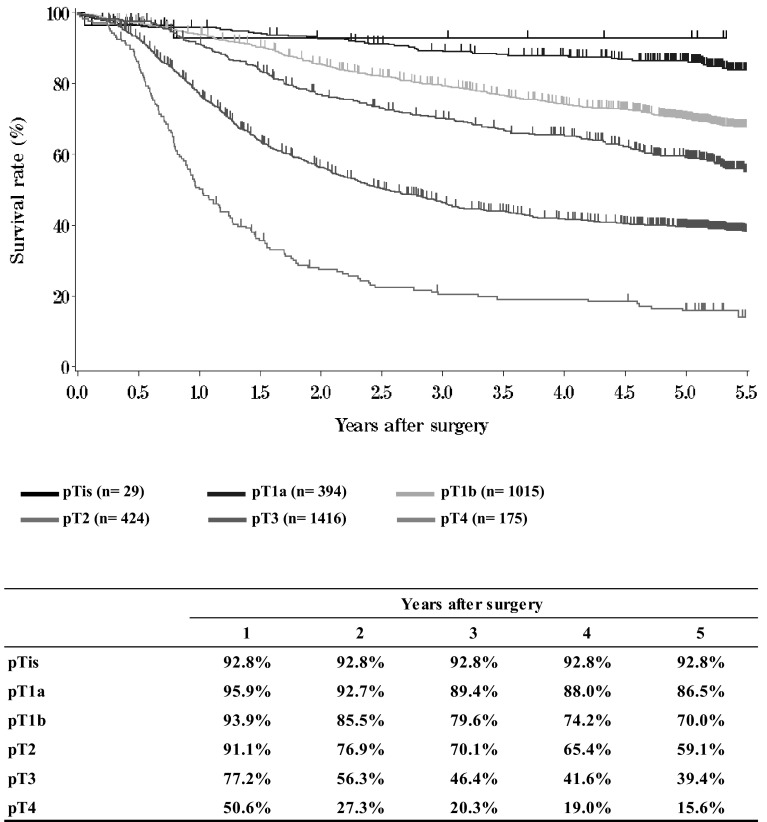
Survival of patients who underwent esophagectomy according to the depth of tumor invasion (JES 10th: pT)
  **Figure****10****Survival of patients who underwent esophagectomy according to the depth of tumor invasion (UICC TNM 6th: pT)**

**Fig. 10:**
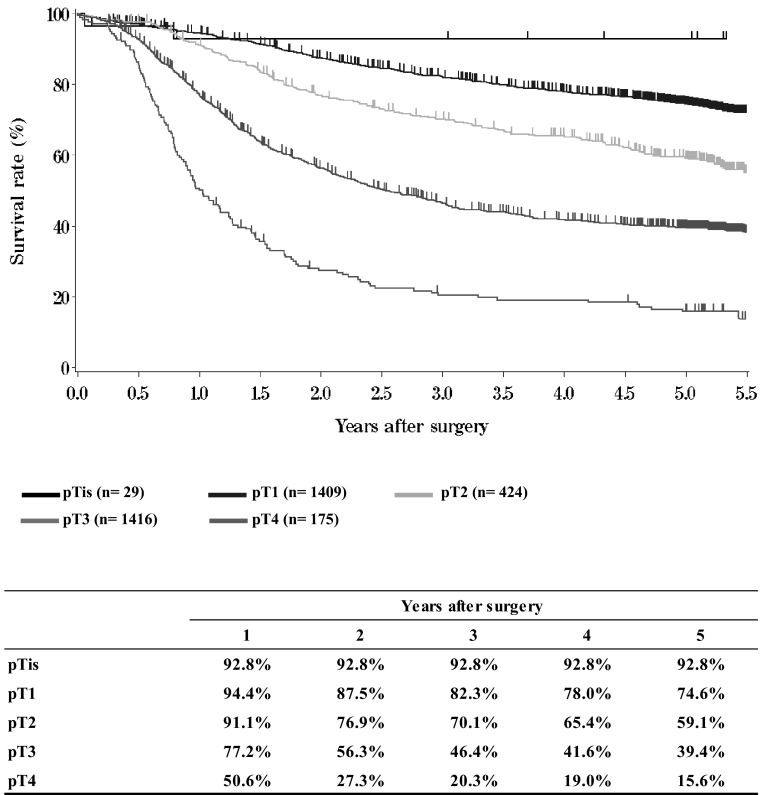
Survival of patients who underwent esophagectomy according to the depth of tumor invasion (UICC TNM 6th: pT)
  **Figure****11****Survival of patients who underwent esophagectomy according to lymph node metastasis (JES 10th: pN)**

**Fig. 11:**
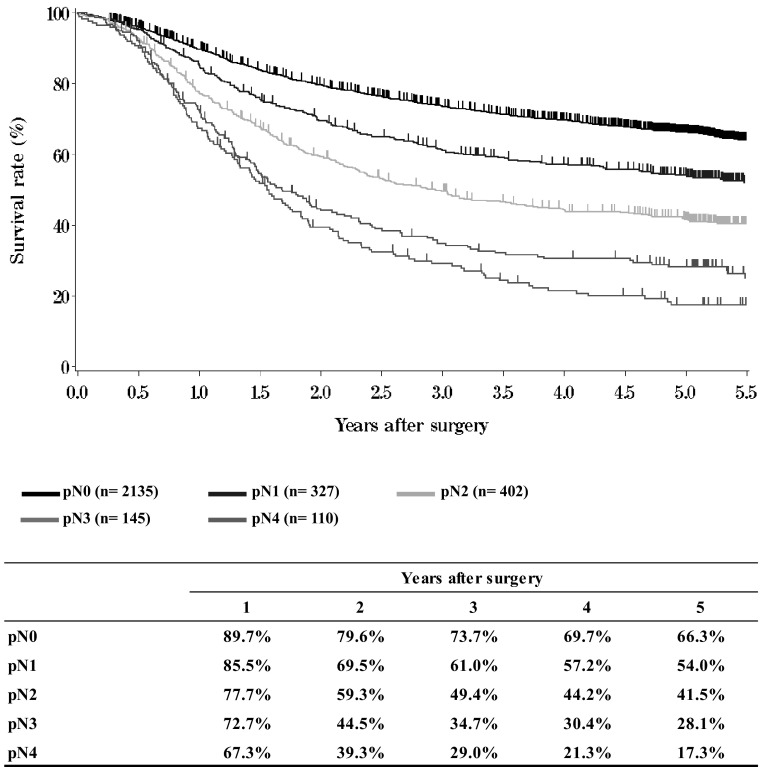
Survival of patients who underwent esophagectomy according to lymph node metastasis (JES 10th: pN)
  **Figure****12****Survival of patients who underwent esophagectomy according to lymph node metastasis (UICC TNM 6th: pN)**

**Fig. 12:**
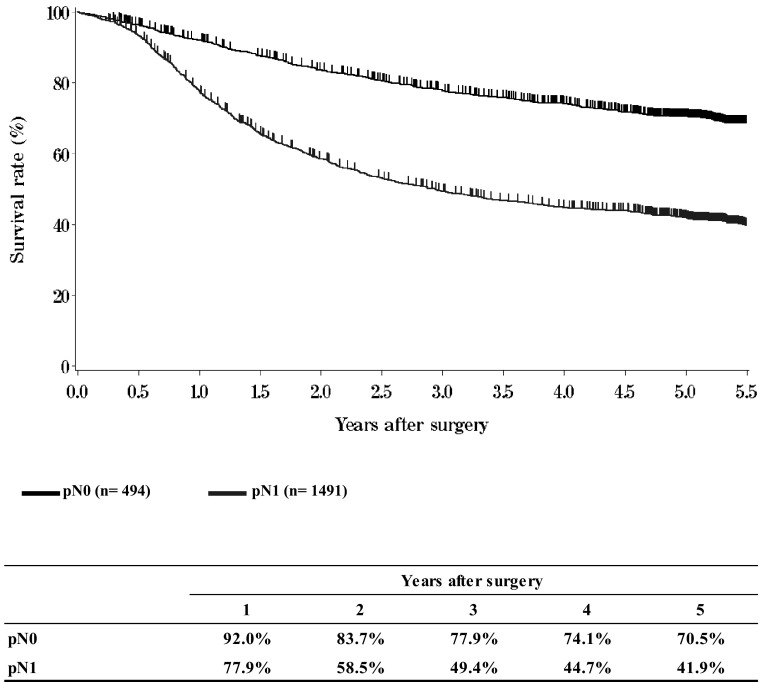
Survival of patients who underwent esophagectomy according to lymph node metastasis (UICC TNM 6th: pN)
  **Figure****13****Survival of patients who underwent esophagectomy according to number of metastatic nodes**

**Fig. 13:**
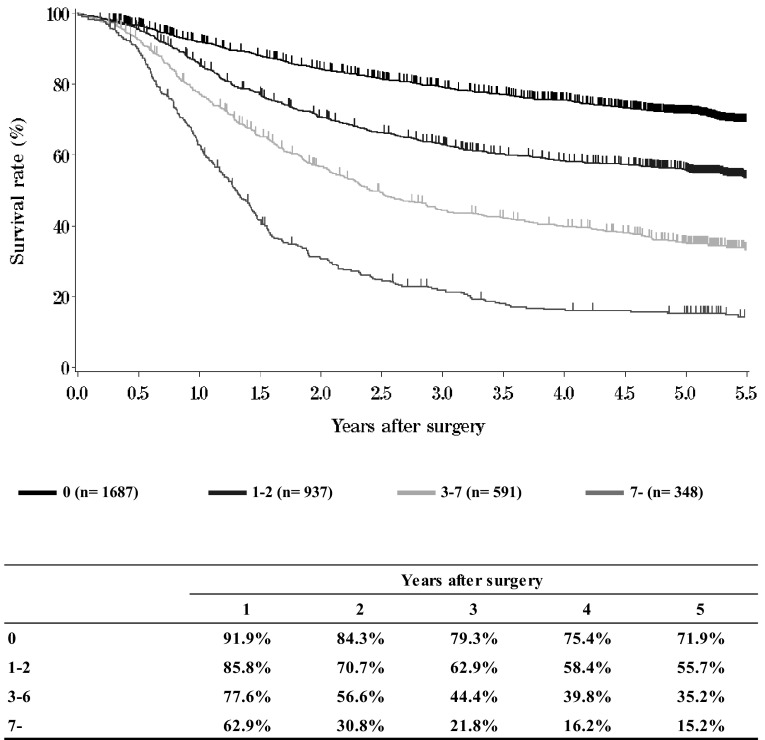
Survival of patients who underwent esophagectomy according to number of metastatic nodes
  **Figure****14****Survival of patients who underwent esophagectomy according to pathological stage (JES 10th)**

**Fig. 14:**
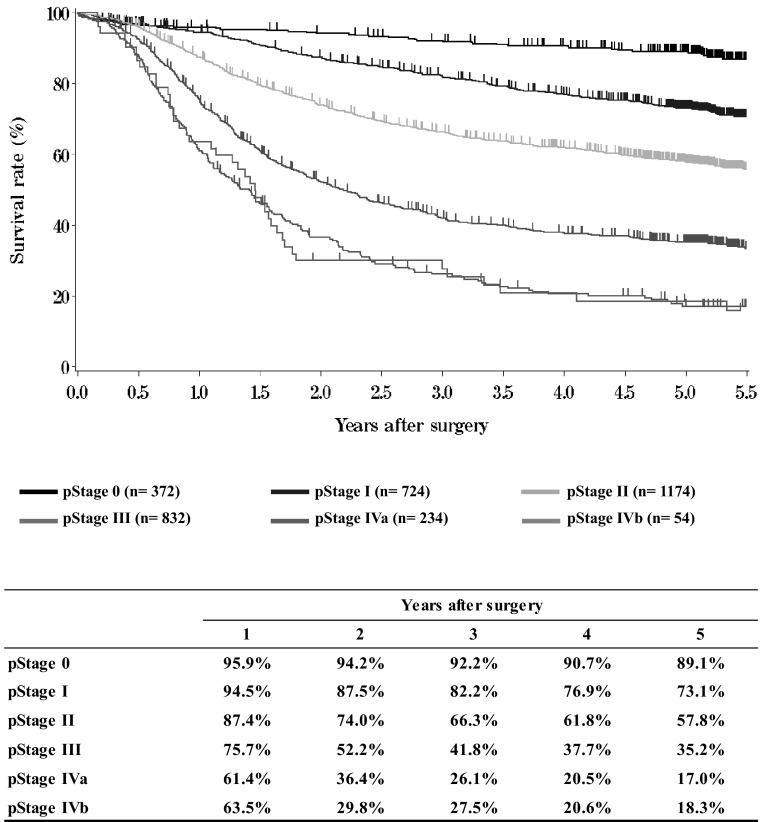
Survival of patients who underwent esophagectomy according to pathological stage (JES 10th)
  **Figure****15****Survival of patients who underwent esophagectomy according to pathological stage (UICC TNM 6th)**

**Fig. 15:**
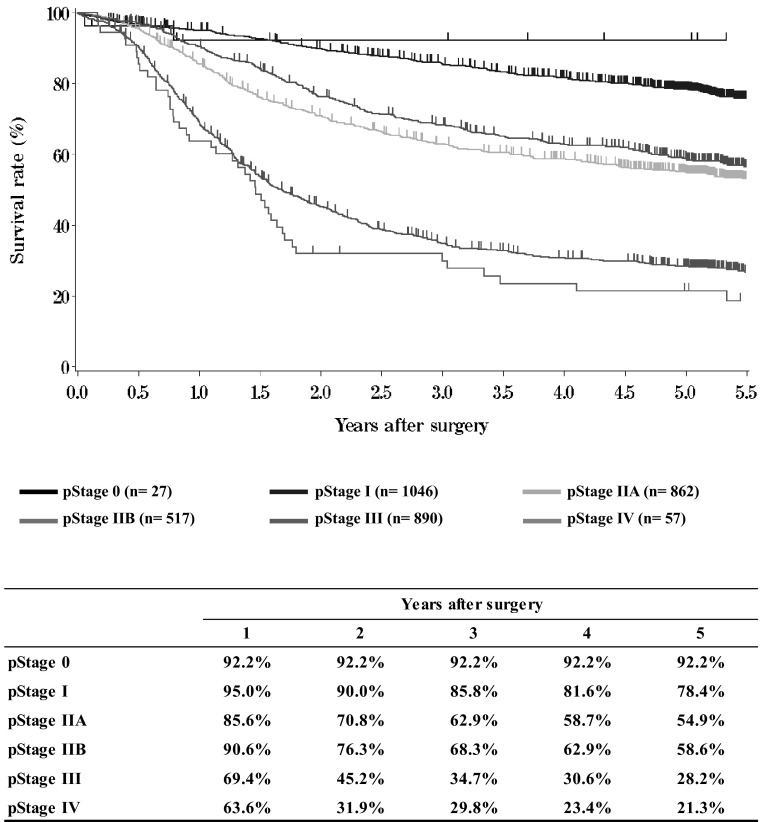
Survival of patients who underwent esophagectomy according to pathological stage (UICC TNM 6th)
  **Figure****16****Survival of patients who underwent esophagectomy according to residual tumor (R)**

**Fig. 16:**
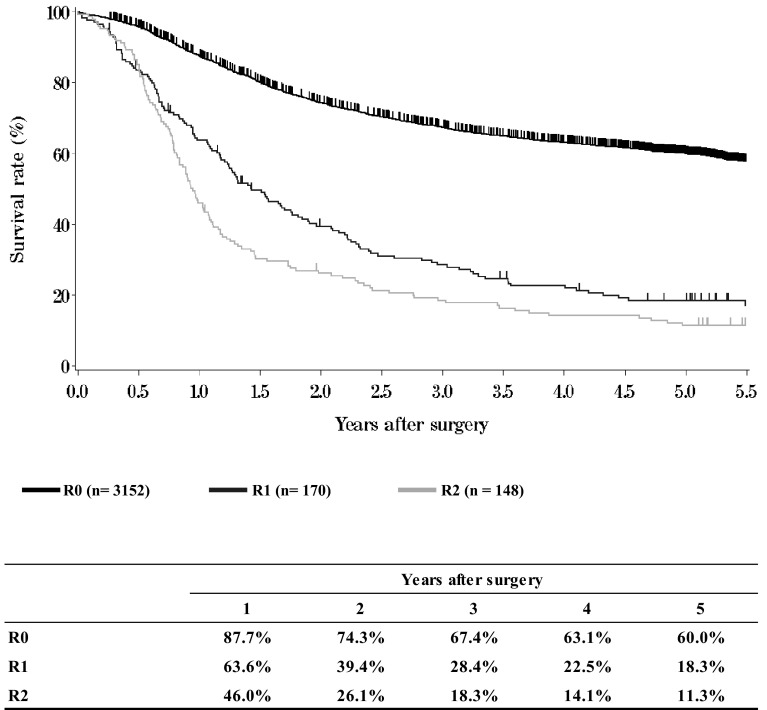
Survival of patients who underwent esophagectomy according to residual tumor (R)

## I. Clinical factors of esophageal cancer patients treated in 2009

### Institution-registered cases in 2009

**Table:** 

Institutions
Aichi Cancer Center
Aizawa Hospital
Akita University Hospital
Aomori Municipal Hospital
Aomori Prefectural Central Hospital
Arao Municipal Hospital
Asahikawa Medical College Hospital
Chiba Cancer Center
Chiba Medical Center
Chiba University Hospital
Chibaken Saiseikai Narashino Hospital
Dokkyo Medical University Hospital
Ehime University Hospital
Foundation for Detection of Early Gastric Carcinoma
Fuchu Hospital
Fujioka General Hospital
Fujisawa Shounandai Hospital
Fujita Health University
Fukui Prefectural Hospital
Fukui University Hospital
Fukuoka Dental College and Dental Hospital
Fukuoka Saiseikai General Hospital?
Fukuoka University Hospital
Fukuoka Wajiro Hospital
Fukushima Medical University Hospital
Fukuyama City Hopital
Gifu Prefectural General Medical Center
Gifu University Hospital
Gunma Central General Hospital
Gunma Prefectural Cancer Center
Gunma University Hospital
Gunmaken Saiseikai Maebashi Hospital
Hachinohe City Hospital
Hakodate Goryokaku Hospital
Hakodate National Hospital
Hamamatsu University School of Medicine, University Hospital
Handa City Hospital
Hannan Chuo Hospital
Heartlife Hospital
Higashiosaka City General Hospital
Hino Memorial Hospital
Hiratsuka City Hospital
Hiratsuka Kyosai Hospital
Hirosaki University Hospital
Hiroshima City Asa Hospital
Hiroshima University Research Institute for Radiation Biology Medicine
Hitachi General Hospital
Hofu Institute of Gastroenterology
Hokkaido Kin-Ikyo Chuo Hospital
Hokkaido University Hospital
Hyogo Cancer Center
Hyogo College of Medicine
Hyogo Prefectural Nishinomiya Hospital
Ibaraki Prefectural Central Hospital
Iizuka Hospital
Imazu Surgical Clinic
Inazawa City Hospital
Internatinal University of Health and Welfare Hospital
Isehara Kyodo Hospital
Ishikawa Prefectural Central Hospital
Iwakuni Medical Center
Iwate Medical University Hospital
Iwate Prefectural Chubu Hospital
Iwate Prefectural Isawa Hospital
Japanese Red Cross Fukui Hospital
Japanese Red Cross Ishinomaki Hospital
Japanese Red Cross Kyoto Daini Hospital?
Japanese Red Cross Maebashi Hospital
Japanese Red Cross Nagaoka Hospital
Japanese Red Cross Narita Hospital
Japanese Red Cross Nasu Hospital
Jichi Medical University Hospital
Juntendo University Hospital
Juntendo University Shizuoka Hospital
Junwakai Memorial Hospital
Kagawa Rosai Hospital
Kagawa University Hospital
Kagoshima Kenritsu Satsunan Hospital
Kagoshima University Hospital
Kameda General Hospital
Kanagawa Cancer Center
Kanazawa Medical University Hospital
Kanazawa University Hospital
Kansai Medical University Hirakata Hospital
Kansai Rosai Hospital
Kashiwa Kousei General Hospital
Kawakita General Hospital
Kawasaki Medical School Hospital
Kawasaki Medical School Kawasaki Hospital
Kawasaki Municipal Hospital
Kawasaki Municipal Ida Hospital
Keio University Hospital
Keiyukai Sapporo Hospital
Kikuna Memorial Hospital
Kinki Central Hospital
Kinki University Hospital
Kiryu Kosei General Hospital
Kishiwada City Hospital
Kitaakita Municipal Hospital
Kitakyushu Municipal Medical Center
Kitano Hospital
Kitasato University Hospital
Kobe City Medical Center General Hospital
Kobe University Hospital
Kochi University Hospital
Kokura Memorial Hospital
Kumamoto City Hospital
Kumamoto University Hospital
Kurashiki Central Hospital
Kurume General Hospital
Kurume University Hospital
Kuwana West Medical Center
Kyoto University Hospital
Kyushu Central Hospital of the Mutual Aid Association of Public School Teachers
Kyushu University Beppu Hospital
Kyushu University Hospital
Kyushu Medical Center
Machida Municipal Hospital
Matsuda Hospital
Matsushita Memorial Hospital
Matsuyama Red Cross Hospital
Mie University Hospital
Mino City Hospital
Mito Red Cross Hospital
Mitsui Memorial Hospital
Miyazaki Konan Hospital
Murakami General Hospital
Musashimurayama Hospital
Musashino Red Cross Hospital
Nagahama City Hospital
Nagano Red Cross Hospital
Nagaoka Chuo General Hospital
Nagasaki University Hospital
Nagayoshi General Hospital
Nagoya City University Hospital
Nagoya City West Medical Center
Nagoya Daiichi Red Cross Hospital
Nagoya University Hospital
Nara Hospital Kinki University Faculty of Medicine
Nara Medical University Hospital
National Cancer Center Hospital
National Cancer Center Hospital East
National Center for Global Health and Medicine
National Defense Medical College Hospital
National Hospital Organization Beppu Medical Center
National Hospital Organization Chiba-East-Hospital
National Hospital Organization Fukuoka-higashi Medical Center
National Hospital Organization Kure Medical Center
National Hospital Organization Kyoto Medical Center
National Hospital Organization Kyushu Cancer Center
National Hospital Organization Matsumoto National Hospital
National Hospital Organization Nagasaki Medical Center.
National Hospital Organization Nagoya Medical Center
National Hospital Organization Osaka National Hospital
National Hospital Organization Tokyo Medical Center
Niigata Cancer Center Hospital
Niigata City General Hospital
Niigata Prefectural Shibata Hospital
Niigata University Medical and Dental Hospital
Nikko Memorial Hospital
Nippon Medical School Chiba Hokusoh Hospital
Nippon Medical School Hospital
Nippon Medical School Musashi Kosugi Hospital
Nippon Medical School Tama Nagayama Hospital
Nishi-Kobe Medical Center
Nishinomiya Municipal Central Hospital
Numazu City Hospital
Obihiro Kousei General Hospital
Ohta General Hospital Foundation Ohta Nishinouchi Hospital
Oita Red Cross Hospital
Oita University Hospital
Okayama Saiseikai General Hospital
Okayama University Hospital
Onomichi Municipal Hospital
Osaka City General Hospital
Osaka City University Hospital
Osaka Hospital of Japan Seafarers relief Association
Osaka Medical Center for Cancer and Cardiovascular Diseases
Osaka Medical College Hospital
Osaka Police Hospital
Osaka Prefectural Hospital Organization Osaka General Medical Center
Osaka Red Cross Hospital
Osaka University Hospital
Otsu Municipal Hospital
Otsu Red Cross Hospital
Rakusei Hospital
Ryukyu University Hospital
Saga University Hospital
Saga-ken Medical Center Koseikan
Saiseikai Fukushima General Hospital
Saiseikai Hiroshima Hospital
Saiseikai Kyoto Hospital
Saiseikai Utsunomiya Hospital
Saitama City Hospital
Saitama Medical Center Jichi Medical University
Saitama Medical University Hospital
Saitama Medical University Saitama International Medical Center
Saitama Medical University Saitama Medical Center
Saitama Medical Center
Sakai City Medical Center
Saku Central Hospital
Sanin Rosai Hospital
Sano Kousei General Hospital
Sendai City Hospital
Shiga Medical Center for Adults
Shiga University of Medical Science Hospital
Shikoku Cancer Center
Shimada Hospital
Shimane University Hospital
Shimizu Welfare Hospital
Shinshu University Hospital
Shizuoka Cancer Center
Shizuoka City Shizuoka Hospital
Shizuoka General Hospital
Showa University Hospital
Showa University Northern Yokohama Hospital
Showa University Koto-Toyosu Hospital
Social Insurance Omuta Tenryo Hospital
Social Insurance Tagawa Hospital
Yokohama Chuo Hospital
Sonoda Daiichi Hospital
St. Marianna University School of Medical Hospital
St. Luke’s International Hospital
Sugita Genpaku Memorial Obama Municipal Hospital
Suita Municipal Hospital
Takasago Municipal Hospital
Takatsuki Red Cross Hospital
Teikyo University Hospital
Tenri Hospital
The Cancer Institute Hospital of JFCR
The Jikei University Hospital
The Research Center Hospital for Charged Particle Therapy of NIRS
Toho University Omori Medical Center
Toho University Sakura Medical Center
Tohoku Kosai Hospital
Tohoku University Hospital
Tokai University Hachioji Hospital
Tokai University Hospital
Tokai University Tokyo Hospital
Tokushima Municipal Hospital
Tokushima Red Cross Hospital
Tokushima University Hospital
Tokyo Dental College Ichikawa General Hospital
Tokyo Medical and Dental University Hospital
Tokyo Medical University Hospital
Tokyo Medical University Ibaraki Medical Center
Tokyo Metropolitan Cancer and Infectious Center Komagome Hospital
Tokyo Metropolitan Health and Medical Corporation Toshima Hospital
Tokyo University Hospital
Tokyo Women’s Medical University Hospital
Tokyo Women’s Medical University Medical Center East
Tonan Hospital
Tone Chuou Hospital
Tottori Prefectural Central Hospital
Tottori University Hospital
Toyama Prefectural Central Hospital
Toyama University Hospital
Tsuchiura Kyodo Hospital
Tsukuba University Hospital
Tsuruoka Municipal Shonai Hospital
“University Hospital, Kyoto Prefectural University of Medicine”
University of Miyazaki Hospital
Wakayama Medical University Hospital
Yamagata Prefectural and Sakata Municipal Hospital Organization
Yamagata Prefectural Central Hospital
Yamagata Prefectural Shinjo Hospital
Yamagata University Hospital
Yamaguchi University Hospital
Yamaguchi-ken Saiseikai Shimonoseki General Hospital
Yamanashi Prefectural Central Hospital
Yamanashi University Hospital
Yao Municipal Hospital
Yokohama City Municipal Hospital
Yokohama City University Hospital
Yokohama City University Medical Center
Yuri General Hospital

(Total 276 institutions)

## Patient Background

**Table 1:** Age and gender

Age	Male	Female	Unknown	Cases (%)
~29	6	1	0	7 (0.1 %)
30–39	12	6	0	18 (0.3 %)
40–49	121	34	0	155 (2.5 %)
50–59	946	173	1	1120 (17.9 %)
60–69	2332	354	0	2686 (42.9 %)
70–79	1575	270	1	1846 (29.5 %)
80–89	303	75	0	378 (6.0 %)
90~	16	3	0	19 (0.3 %)
Unknown	27	4	0	31 (0.5 %)
Total	5338	920	2	6260 (100 %)

**Table 2:** Primary treatment

Treatments	Cases (%)
Surgery	3943 (63.0 %)
Esophagectomy	3844 (61.8 %)
Palliative	99 (1.2 %)
Chemotherapy/radiotherapy	1383 (22.1 %)
Endoscopic treatment	932 (14.9 %)
Others	2 (0.0 %)
Total	6260 (100 %)

**Table 3:** Tumor location

Location of tumor	Endoscopic treatment (%)	Chemotherapy and/or radiotherapy (%)	Palliative surgery (%)	Esophagectomy (%)	Other (%)	Total (%)
Cervical	18 (1.9 %)	112 (8.1 %)	9 (9.1 %)	137 (3.6 %)	0	276 (4.4 %)
Upper thoracic	105 (11.3 %)	184 (13.3 %)	19 (19.2 %)	437 (11.4 %)	0	745 (11.9 %)
Middle thoracic	511 (54.8 %)	665 (48.1 %)	50 (50.5 %)	1778 (46.3 %)	1 (50.0 %)	3005 (48.0 %)
Lower thoracic	243 (26.1 %)	325 (23.5 %)	18 (18.2 %)	1147 (29.8 %)	0	1733 (27.7 %)
E > G	40 (4.3 %)	38 (2.7 %)	2 (2.0 %)	245 (6.4 %)	0	325 (5.2 %)
E = G	5 (0.5 %)	6 (0.4 %)	0	41 (1.1 %)	0	52 (0.8 %)
G > E	0	2 (0.1 %)	0	34 (0.9 %)	0	36 (0.6 %)
Unknown	10 (1.1 %)	51 (3.7 %)	1 (1.0 %)	25 (0.7 %)	1 (50.0 %)	88 (1.4 %)
Total	932 (100 %)	1383 (100 %)	99 (100 %)	3844 (100 %)	2 (100 %)	6260 (100 %)

*E* esophageal, *G* gastric

**Table 4:** Histologic types of biopsy specimens

Histologic types	Cases (%)
Squamous cell carcinoma	5665 (90.5 %)
Squamous cell carcinoma	3827 (61.1 %)
Well differentiated	354 (5.7 %)
Moderately differentiated	1140 (18.2 %)
Poorly differentiated	344 (5.5 %)
Adenocarcinoma	296 (4.7 %)
Adenosquamous carcinoma	13 (0.2 %)
Mucoepidermoid carcinoma	1 (0.0 %)
Basaloid carcinoma	22 (0.4 %)
Neuroendocrine cell carcinoma	14 (0.2 %)
Undifferentiated carcinoma	10 (0.2 %)
Malignant melanoma	7 (0.1 %)
Carcinosarcoma	17 (0.3 %)
Other tumors	28 (0.4 %)
Unknown	187 (3.0 %)
Total	6260 (100 %)

**Table 5:** Depth of tumor invasion, cT (UICC TNM 6th)

cT	Cases (%)
cTX	29 (0.5 %)
cT0	11 (0.2 %)
cTis	157 (2.5 %)
cT1	359 (5.7 %)
cT1a	650 (10.4 %)
cT1b	1134 (18.1 %)
cT2	868 (13.9 %)
cT3	2252 (36.0 %)
cT4	701 (11.2 %)
Unknown	99 (1.6 %)
Total	6260 (100 %)

**Table 6:** Lymph node metastasis, cN (UICC TNM 6th)

cN	Cases (%)
cNX	72 (1.2 %)
cN0	2920 (46.6 %)
cN1	3157 (50.4 %)
Unknown	111 (1.8 %)
Total	6260 (100 %)

**Table 7:** Distant metastasis, cM (UICC TNM 6th)

cM	Cases (%)
cMX	57 (0.9 %)
cM0	5295 (84.6 %)
cM1	223 (3.6 %)
cM1a	141 (2.3 %)
cM1b	466 (7.4 %)
Total	6260 (100 %)

**Table 8:** Clinical Stage (UICC TNM 6th)

Location of tumor	Endoscopic treatment (%)	Chemotherapy and/or radiotherapy (%)	Palliative surgery (%)	Esophagectomy (%)	Other (%)	Total (%)
0	131 (14.1 %)	6 (0.4 %)	1 (1.0 %)	13 (0.3 %)	0	151 (2.4 %)
I	658 (70.6 %)	152 (11.0 %)	2 (2.0 %)	964 (25.1 %)	0	1776 (28.4 %)
IIA	6 (0.6 %)	125 (9.0 %)	7 (7.1 %)	717 (18.7 %)	0	855 (13.7 %)
IIB	7 (0.8 %)	98 (7.1 %)	2 (2.0 %)	555 (14.4 %)	0	662 (10.6 %)
III	29 (3.1 %)	452 (32.7 %)	62 (62.6 %)	1243 (32.3 %)	1 (50.0 %)	1787 (28.5 %)
IV	10 (1.1 %)	139 (10.1 %)	7 (7.1 %)	44 (1.1 %)	0	200 (3.2 %)
IVA	5 (0.5 %)	53 (3.8 %)	1 (1.0 %)	81 (2.1 %)	0	140 (2.2 %)
IVB	18 (1.9 %)	265 (19.2 %)	12 (12.1 %)	156 (4.1 %)	0	451 (7.2 %)
Unknown	68 (7.3 %)	93 (6.7 %)	5 (5.1 %)	71 (1.8 %)	1 (50.0 %)	238 (3.8 %)
Total	932 (100 %)	1383 (100 %)	99 (100 %)	3844 (100 %)	2 (100 %)	6260 (100 %)

## II. Results of endoscopically treated patients in 2009

**Table 9:** Details of endoscopic treatment

Treatment details	Cases (%)
EMR	201 (21.6 %)
EMR + ESD	11 (1.2 %)
EMR + YAG laser	7 (0.8 %)
ESD	607 (65.1 %)
ESD + other treatment	7 (0.8 %)
PDT	2 (0.2 %)
PDT + YAG laser	2 (0.2 %)
YAG laser	10 (1.1 %)
Esophageal stenting	70 (7.5 %)
Esophageal stenting + tracheal stenting	2 (0.2 %)
Tracheal stenting	4 (0.4 %)
Others	5 (0.5 %)
Unknown	4 (0.4 %)
Total	753 (100 %)

*EMR* endoscopic mucosal resection, *ESD* endoscopic submucosal dissection, *YAG*: yttrium aluminum garnet, *PDT* photodynamic therapy

**Table 10:** Complications of EMR/ESD

Complications of EMR/ESD	Cases (%)
None	766 (91.8 %)
Perforation	16 (1.9 %)
Bleeding	2 (0.2 %)
Mediastinitis	0
Stenosis	42 (5.0 %)
Others	7 (0.8 %)
Unknown	1 (0.1 %)
Total	834 (100 %)

**Table 11:** Pathological depth of tumor invasion of EMR/ESD specimens

Pathological depth of tumor invasion	Cases (%)
pTX	1 (0.1 %)
pT0	5 (0.6 %)
pTis	166 (19.9 %)
pT1a	507 (60.9 %)
pT1b	86 (10.3 %)
pT2	0
Unknown	68 (8.2 %)
Total	833 (100 %)

## III. Results in patients treated with chemotherapy and/or radiotherapy in 2009

**Table 12:** Dose of radiation (non-surgically treated cases)

Dose of radiation (Gy)	Definitive		Palliative (%)	Recurrence (%)	Others (%)	Unknown (%)	Total (%)
	Radiation alone (%)	With chemotherapy (%)					
–29	5 (4.1 %)	18 (2.3 %)	23 (11.0 %)	2 (6.1 %)	0	1 (5.6 %)	49 (4.1 %)
30–39	1 (0.8 %)	15 (1.9 %)	25 (11.9 %)	3 (9.1 %)	1 (7.1 %)	0	45 (3.8 %)
40–49	11 (8.9 %)	40 (5.1 %)	31 (14.8 %)	9 (27.3 %)	8 (57.1 %)	1 (5.6 %)	100 (8.4 %)
50–59	24 (19.5 %)	199 (25.3 %)	47 (22.4 %)	8 (24.2 %)	2 (14.3 %)	1 (5.6 %)	281 (23.7 %)
60–69	74 (60.2 %)	493 (62.6 %)	81 (38.6 %)	9 (27.3 %)	2 (14.3 %)	15 (83.3 %)	674 (56.8 %)
70-	6 (7.2 %)	8 (2.1 %)	2 (0.0 %)	0	0	0	16 (2.2 %)
Unknown	2 (1.6 %)	15 (1.9 %)	1 (0.5 %)	2 (6.1 %)	1 (7.1 %)	0	21 (1.8 %)
Total	123 (100 %)	788 (100 %)	210 (100 %)	33 (100 %)	14 (100 %)	18 (100 %)	1186 (100 %)
Median (min–max)	60.0 (6.0–120.0)	60.0 (2.0–124.0)	54.0 (2.0–95.4)	50.0 (20.0–66.0)	40.0 (36.0–60.0)	60.0 (2.0–61.2)	60.0 (2.0–124.0)

**Table 13:** Dose of radiation (surgically treated cases)

Dose of radiation (Gy)	Preoperative radiation (%)	Postoperative radiation (%)
–29	3 (1.4 %)	1 (1.4 %)
30–39	54 (24.4 %)	2 (2.7 %)
40–49	132 (59.7 %)	21 (28.4 %)
50–59	9 (4.1 %)	18 (24.3 %)
60–69	15 (6.8 %)	27 (36.5 %)
70-	0	0 (1.1 %)
Unknown	8 (3.6 %)	5 (6.8 %)
Total	221 (100 %)	74 (100 %)
Median (min–max)	40.0 (15.0–66.0)	50.4 (4.0–64.0)

## IV. Results in patients who underwent esophagectomy in 2009

**Table 14:** Treatment modalities of esophagectomy

Treatments	Cases (%)
Esophagectomy	1630 (42.4 %)
Esophagectomy + radiotherapy	65 (1.7 %)
Esophagectomy + chemoradiotherapy	655 (17.0 %)
Esophagectomy + chemoradiotherapy + endoscopic treatment	16 (0.4 %)
Esophagectomy + chemoradiotherapy + other treatment	2 (0.1 %)
Esophagectomy + radiotherapy + endoscopic treatment	3 (0.1 %)
Esophagectomy + radiotherapy + other treatment	1 (0.0 %)
Esophagectomy + chemotherapy	1385 (36.0 %)
Esophagectomy + chemotherapy + endoscopic treatment	8 (0.2 %)
Esophagectomy + chemotherapy + other treatment	2 (0.1 %)
Esophagectomy + endoscopic treatment	77 (2.0 %)
Total	3844 (100 %)

**Table 15:** Tumor location

Locations	Cases (%)
Cervical	137 (3.6 %)
Upper thoracic	437 (11.4 %)
Middle thoracic	1778 (46.3 %)
Lower thoracic	1147 (29.8 %)
E > G	245 (6.4 %)
E = G	41 (1.1 %)
G > E	34 (0.9 %)
Unknown	25 (0.7 %)
Total lesions	3844 (100 %)

**Table 16:** Approaches to tumor resection

Approaches	Cases (%)
Cervical approach	132 (3.4 %)
Right thoracotomy	3239 (84.3 %)
Left thoracotomy	66 (1.7 %)
Left thoracoabdominal approach	49 (1.3 %)
Laparotomy	148 (3.9 %)
Transhiatal thoracic esophagectomy	52 (1.4 %)
Transhiatal lower esophagectomy	92 (2.4 %)
Sternotomy	2 (0.1 %)
Others	32 (0.8 %)
Unknown	32 (0.8 %)
Total	3844 (100 %)

**Table 17:** Video-assisted surgery

Video-assisted surgery	Cases (%)
None	2549 (66.3 %)
Thoracoscopy	554 (14.4 %)
Laparoscopy	124 (3.2 %)
Thoracoscopy + laparoscopy	388 (10.1 %)
Mediastinoscopy	26 (0.7 %)
Thoracoscopy + laparoscopy + mediastinoscopy	4 (0.1 %)
Thoracoscopy + other	11 (0.3 %)
Laparoscopy + mediastinoscopy	5 (0.1 %)
Others	17 (0.4 %)
Unknown	166 (4.3 %)
Total	3844 (100 %)

**Table 18:** Fields of lymph node dissection according to the location of the tumor

Field of lymphadenectomy	Cervical	Upper thoracic	Middle thoracic	Lower thoracic	E > G	E = G	G > E	Unknown	Total
None	9 (6.6 %)	21 (4.8 %)	76 (4.3 %)	39 (3.4 %)	5 (2.0 %)	4 (9.8 %)	1 (2.9 %)	6 (75.0 %)	161 (4.2 %)
C	51 (37.2 %)	5 (1.1 %)	13 (0.7 %)	3 (0.3 %)	0 (0.0 %)	0	0	1 (12.5 %)	73 (1.9 %)
C + UM	20 (14.6 %)	5 (1.1 %)	0	0	0	0	0	0	25 (0.7 %)
C + UM + MLM	7 (5.1 %)	9 (2.1 %)	45 (2.5 %)	18 (1.6 %)	2 (0.8 %)	0	0	0 (0.0 %)	81 (2.1 %)
C + UM + MLM + A	35 (25.5 %)	286 (65.4 %)	935 (52.6 %)	435 (37.9 %)	29 (11.8 %)	3 (7.3 %)	1 (2.9 %)	8 (100.0 %)	1732 (45.1 %)
C + UM + MLM + A + other	2 (1.5 %)	4 (0.9 %)	0	1 (0.1 %)	0	0	0	0	7 (0.2 %)
C + UM + A	0 (0.0 %)	1 (0.2 %)	1 (0.1 %)	0	0	0	0	0	2 (0.1 %)
C + MLM + A	0	2 (0.5 %)	4 (0.2 %)	4 (0.3 %)	0	0	0	0	10 (0.3 %)
C + A	5 (3.6 %)	1 (0.2 %)	3 (0.2 %)	3 (0.3 %)	0	0	0	1 (12.5 %)	13 (0.3 %)
UM	0	5 (1.1 %)	4 (0.2 %)	5 (0.4 %)	0	0	0	0	14 (0.4 %)
UM + MLM	2 (1.5 %)	10 (2.3 %)	17 (1.0 %)	13 (1.1 %)	2 (0.8 %)	0	0	1 (12.5 %)	45 (1.2 %)
UM + MLM + A	0	64 (14.6 %)	584 (32.8 %)	485 (42.3 %)	81 (33.1 %)	9 (22.0 %)	5 (14.7 %)	2 (25.0 %)	1230 (32.0 %)
UM + MLM + A + other	0	0 (0.0 %)	1 (0.1 %)	0	0	0	0	0	1 (0.0 %)
UM + A	0	1 (0.2 %)	3 (0.2 %)	3 (0.3 %)	0	0	0	0	7 (0.2 %)
MLM	0	3 (0.7 %)	13 (0.7 %)	5 (0.4 %)	3 (1.2 %)	0	0	0 (0.0 %)	24 (0.6 %)
MLM + A	1 (0.7 %)	12 (2.7 %)	50 (2.8 %)	104 (9.1 %)	91 (37.1 %)	16 (39.0 %)	9 (26.5 %)	3 (37.5 %)	286 (7.4 %)
A	1 (0.7 %)	5 (1.1 %)	20 (1.1 %)	24 (2.1 %)	30 (12.2 %)	9 (22.0 %)	18 (52.9 %)	0	107 (2.8 %)
Unknown	4 (2.9 %)	3 (0.7 %)	9 (0.5 %)	5 (0.4 %)	2 (0.8 %)	0	0	3 (37.5 %)	26 (0.7 %)
Total	137 (100 %)	437 (100 %)	1778 (100 %)	1147 (100 %)	245 (100 %)	41 (100 %)	34 (100 %)	25 (100 %)	3844 (100 %)

*C* bilateral cervical nodes, *UM* upper mediastinal nodes, *MLM* middle-lower mediastinal nodes, *A* abdominal nodes

**Table 19:** Reconstruction route

Reconstruction route	Cases (%)
None	48 (1.2 %)
Subcutaneous	323 (8.4 %)
Retrosternal	1422 (37.0 %)
Intrathoracic	446 (11.6 %)
Posterior mediastinal	1491 (38.8 %)
Cervical	49 (1.3 %)
Others	36 (0.9 %)
Unknown	29 (0.8 %)
Total	3844 (100 %)

**Table 20:** Organs used for reconstruction

Organs used for reconstruction	Cases (%)
None	51 (1.3 %)
Whole stomach	102 (2.6 %)
Gastric tube	3234 (81.6 %)
Jejunum	213 (5.4 %)
Free jejunum	88 (2.2 %)
Colon	153 (3.9 %)
Free colon	12 (0.3 %)
Skin graft	0 (0.0 %)
Others	93 (2.3 %)
Unknown	18 (0.5 %)
Total organs	3964 (100 %)
Total cases	3844

**Table 21:** Histological classification

Histological classification	Cases (%)
Squamous cell carcinoma	3300 (86.7 %)
Squamous cell carcinoma	685 (18.0 %)
Well differentiated	653 (17.2 %)
Moderately differentiated	1521 (40.0 %)
Poorly differentiated	441 (11.6 %)
Adenocarcinoma	222 (5.8 %)
Adenosquamous cell carcinoma	35 (0.9 %)
Adenoid cystic carcinoma	1 (0.0 %)
Basaloid carcinoma	56 (1.5 %)
Neuroendocrine cell carcinoma	17 (0.4 %)
Undifferentiated carcinoma	10 (0.3 %)
Other carcinoma	9 (0.2 %)
Carcinosarcoma	21 (0.6 %)
Malignant melanoma	11 (0.3 %)
GIST	1 (0.0 %)
Other	46 (1.2 %)
Unknown	78 (2.0 %)
Total	3807 (100 %)

**Table 22:** Depth of tumor invasion, pT (JES 10th)

pT category	Cases (%)
pTX	24 (0.6 %)
pT0	94 (2.4 %)
pTis	29 (0.8 %)
pT1a	422 (11.0 %)
pT1b	1065 (27.7 %)
pT2	454 (11.8 %)
pT3	1518 (39.5 %)
pT4	127 (3.3 %)
pT4a	27 (0.7 %)
pT4b	30 (0.8 %)
Unknown	54 (1.4 %)
Total	3844 (100 %)

**Table 23:** Pathological grading of lymph node metastasis, pN (JES 10th)

Lymph node metastasis	Cases (%)
pN0	2270 (59.1 %)
pN1	492 (12.8 %)
pN2	584 (15.2 %)
pN3	225 (5.9 %)
pN4	185 (4.8 %)
Unknown	88 (2.3 %)
Total	3844 (100 %)

**Table 24:** Numbers of the metastatic nodes

Numbers of lymph node metastasis	Cases (%)
0	1779 (46.3 %)
1-2	985 (25.6 %)
3-6	640 (16.6 %)
7-	376 (9.8 %)
Unknown	64 (1.7 %)
Total	3844 (100 %)

**Table 25:** Pathological findings of distant organ metastasis, pM (JES 10th)

Distant metastasis	Cases (%)
pMX	53 (1.4 %)
pM0	3733 (97.1 %)
pM1	58 (1.5 %)
Total	3844 (100 %)

**Table 26:** Residual tumor, R

Residual tumor	Cases (%)
RX	156 (4.1 %)
R0	3345 (87.0 %)
R1	187 (4.9 %)
R2	156 (4.1 %)
Total	3844 (100 %)

**Table 27:** Causes of death

Cause of death	Cases (%)
Death due to recurrence	1139 (72.8 %)
Death due to other cancer	65 (4.2 %)
Death due to other disease (rec +)	44 (2.8 %)
Death due to other disease (rec-)	179 (11.4 %)
Death due to other disease (rec?)	7 (0.4 %)
Operative death*	39 (2.5 %)
Postoperative hospital death**	40 (2.6 %)
Unknown	52 (3.3 %)
Total of death cases	1565 (100 %)

rec recurrence

* Operative death means death within 30 days after operation in or out of hospital

** Hospital death is defined as death during the same hospitalization, regardless of department at time of death

Operative mortality after esophagectomy: 1.01 %

Hospital mortality after esophagectomy: 4.76 %

